# Supplementary material for: Natural Extracts to Augment Energy Expenditure as a Complementary Approach to Tackle Obesity and Associated Metabolic Alterations
Source: Biomolecules. 2021 Mar 10;11(3):412. doi: 10.3390/biom11030412 (PMC7999034; doi:10.3390/biom11030412)
Supplement: Supplementary file 1 [file biomolecules-11-00412-s001.pdf]

biomolecules

Supplementary material

Natural extracts to augment energy expenditure as a complementary approach to tackle obesity and associated metabolic alterations.

Marina Reguero[1,2,*], Marta Gómez de Cedrón[1,*,#], Guillermo Reglero[3], José Carlos Quintela[2] and Ana Ramírez de Molina[1,#]

1 Molecular Oncology Group. Precision Nutrition and Health, IMDEA Food Institute, CEI UAM + CSIC, Ctra. de Cantoblanco 8, 28049, Madrid, Spain
2 NATAC BIOTECH, Electronica 7, 28923 Alcorcón, Madrid, Spain
3 Production and Characterization of Novel Foods Department, Institute of Food Science Research CIAL, CEI UAM + CSIC, 28049, Madrid, Spain
# Correspondence: Ana Ramírez de Molina (ana.ramirez@imdea.org) and Marta Gómez de Cedrón (marta.gomezdecedron@imdea.org)
* Equal contribution

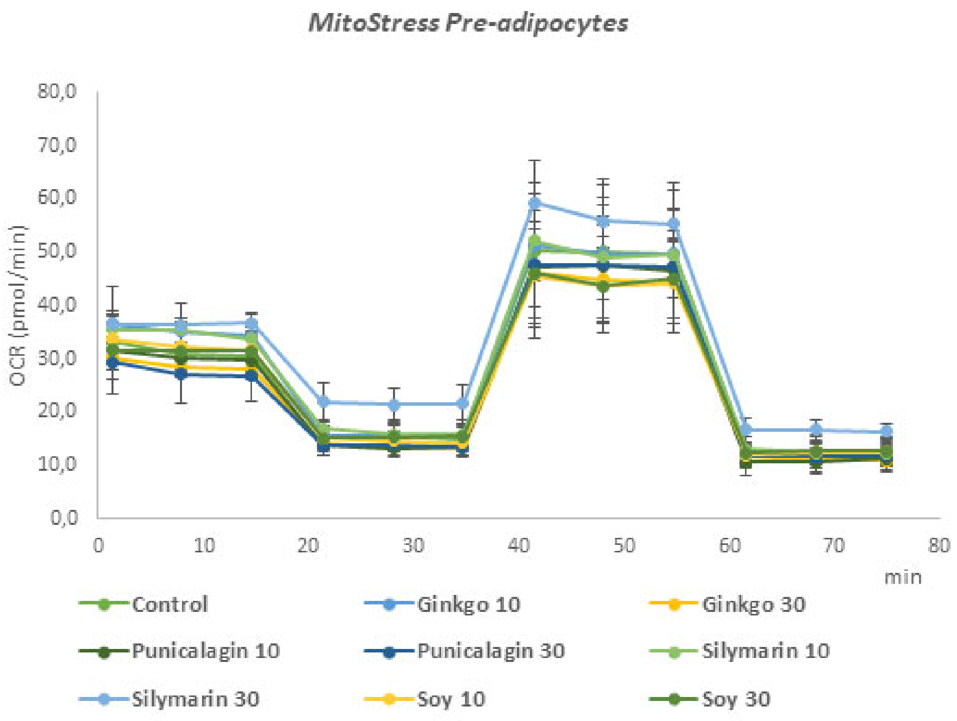

Figure S1. Effect of the extracts on mitochondrial oxidative phosphorylation of humannon-differentiated pre-adipocytes.

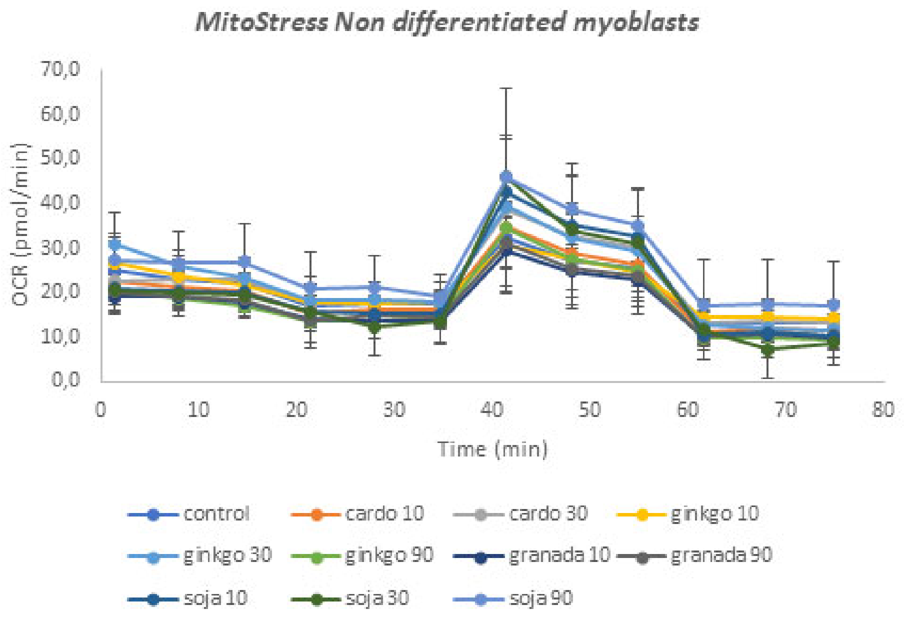

Figure S2. Effect of the extracts on mitochondrial oxidative phosphorylation of human non differentiated myoblasts.

Table S1. List of Taqman probes used in the study.

GENES	Thermofisher probe's code
ADRB1	Hs02330048_s1
AGT	Hs01586213_m1
LEPR	Hs00174497_m1
LPL	Hs00173425_m1
MTHFR	Hs01114487_m1
PPARγ	Hs01115513_m1
TCF7L2	Hs01009044_m1
UCP2	Hs01075227_m1
ACE	Hs00174179_m1
CLOCK	Hs00231857_m1
COL5A1	Hs00609133_m1
HFE	Hs05045803_s1
HIF1A	Hs00153153_m1
PPARA	Hs00947536_m1
PPARGC1A	Hs00173304_m1
AHR	Hs00169233_m1
BNC2	Hs00417700_m1
DLGAP1	Hs00191052_m1
COMT	Hs00241349_m1
IL4-R	Hs00965056_m1
HLA-DQA1	Hs03007426_mH
MCM6	Hs00962418_m1
ABCA1	Hs01059137_m1
FTO	Hs01057139_g1
MC4R	Hs00271877_s1
TMEM18	Hs00894216_m1
GNPDA2	Hs00385682_m1
KCTD15	Hs00225337_m1
ETV5	Hs00927557_m1
NPY	Hs00173470_m1
APOA5	Hs00364830_m1
GCKR	Hs00386984_m1
CD36	Hs00354519_m1
B2M	Hs00187842_m1
GAPDH	Hs02786624_g1

Table S2. List of primers used in the study.

GEN	FORWARD PRIMER	bp	REVERSE PRIMER	bp	SPECIE
MYOG	TCAACCAGGAGGAGCGTGA	19	TCTGTAGGGTCAGCCGTGAG	20	human
MEF2	AACAAAGCCCTCAGCAGGT	19	ACTTGCACAGCTCAGTTCCC	20	human
UCP1	ACAGAAGGGCGGATGAAACT	20	CTTGCTTCCTAAACTAGGTGCTG	23	human
TFAM	ATGGCGTTTCTCCGAAGCAT	20	TCCGCCCTATAAGCATCTTGA	21	human
PPARG	ACCAAAGTGCAATCAAAGTGGA	22	ATGAGGGAGTTGGAAGGCTCT	21	human
UCP3	AAGGTCCGATTTCAGGCCAG	20	GCGATGGTTCTGTAGGCGTC	20	human
C/EBPa	TATAGGCTGGGCTTCCCCTT	20	AGCTTTCTGGTGTGACTCGG	20	human
ATP2A1	AAACCACGGAGGAATGTTTGG	21	AGCTCATTGAGGCCGTATTTC	21	human
MYH2	AGAAACTTCGCATGGACCTAGA	22	CCAAGTGCCTGTTCATCTTCA	21	human
FABP4	ACTGGGCCAGGAATTTGACG	20	CTCGTGGAAGTGACGCCTT	19	human
Cidea	TTATGGGATCACAGACTAAGCGA	23	TGCTCCTGTCATGGTTGGAGA	21	human
Ckmt2	GACCCCGTCATCAAACTAAGAC	22	AGCACGTAATGCTCGTCGAAC	21	human
Lep	TGCCTTCCAGAAACGTGATCC	21	CTCTGTGGAGTAGCCTGAAGC	21	human
PRDM16	CGATGCCGACTTTTGGGAAG	20	GTGGAGAGGAGTGTCTTCGG	20	human
PGC1a	ATTGGAGCCCCATGGATGAA	20	GCGGCTGTTACTCTCTCTCC	20	human
CPT1A	CTCAGTGGGAGCGGATGTTT	20	TGCTGTCTCTCATGTGCTGG	20	human
LDLR	CAGCTACCCCTCGAGACAGA	20	CACTGTCCGAAGCCTGTTCT	20	human
IRS1	GCAACCAGAGTGCCAAAGTG	20	GCACTGGGTGTTGAGGAGAA	20	human
FASN	TATGAAGCCATCGTGGACGG	20	GAAGAAGGAGAGCCGGTTGG	20	human
UCP2	GACCATTGCCCGAGAGGAAG	20	GAAGTGGCAAGGGAGGTCAT	20	human
FNDC5	CGTGGTCCTGTTCATGTGGG	20	GGTCTTGCCCTCACCTTGCT	20	human
BDNF	TAACGGCGGCAGACAAAAAGA	21	TGCACTTGGTCTCGTAGAAGTAT	23	human
IL6	CCTGAACCTTCCAAAGATGGC	21	TTCACCAGGCAAGTCTCCTCA	21	human
SIRT1	TGTGTCATAGGTTAGGTGGTGA	22	AGCCAATTCTTTTTGTGTTCGTG	23	human
B2M	GATGAGTATGCCTGCCGTGT	20	TGCGGCATCTTCAAACCTCC	20	human
